# Estimating heritability of complex traits from genome-wide association studies using IBS-based Haseman–Elston regression

**DOI:** 10.3389/fgene.2014.00107

**Published:** 2014-04-30

**Authors:** Guo-Bo Chen

**Affiliations:** Queensland Brain Institute, The University of QueenslandSt. Lucia, QLD, Australia

**Keywords:** Haseman–Elston regression, GWAS, identity by state, variance component, missing heritability, case-control, mixed linear model, REML

## Abstract

Exploring heritability of complex traits is a central focus of statistical genetics. Among various previously proposed methods to estimate heritability, variance component methods are advantageous when estimating heritability using markers. Due to the high-dimensional nature of data obtained from genome-wide association studies (GWAS) in which genetic architecture is often unknown, the most appropriate heritability estimator model is often unclear. The Haseman–Elston (HE) regression is a variance component method that was initially only proposed for linkage studies. However, this study presents a theoretical basis for a modified HE that models linkage disequilibrium for a quantitative trait, and consequently can be used for GWAS. After replacing identical by descent (IBD) scores with identity by state (IBS) scores, we applied the IBS-based HE regression to single-marker association studies (scenario I) and estimated the variance component using multiple markers (scenario II). In scenario II, we discuss the circumstances in which the HE regression and the mixed linear model are equivalent; the disparity between these two methods is observed when a covariance component exists for the additive variance. When we extended the IBS-based HE regression to case-control studies in a subsequent simulation study, we found that it provided a nearly unbiased estimate of heritability, more precise than that estimated via the mixed linear model. Thus, for the case-control scenario, the HE regression is preferable. GEnetic Analysis Repository (GEAR; http://sourceforge.net/p/gbchen/wiki/GEAR/) software implemented the HE regression method and is freely available.

## Introduction

So-called “missing heritability” can occur due to various reasons, such as small sample size, underrepresented variant spectrum, experimental design, and improper methodological assumptions (Manolio et al., 2009). Because of the high-dimensional nature of genome-wide association study (GWAS) data, in which the number of markers (*M*) is far greater than the number of individuals (*N*), estimating heritability is difficult. For instance, if the statistical power is insufficient, variants associated with a small effect may not be captured under a stringent *p*-value threshold (~ 10^−8^). This obstacle can be partially bypassed by implementing the mixed linear model, which uses the genetic relationship between individuals estimated from single nucleotide polymorphism (SNP) markers *in lieu* of fitting hundreds of thousands of markers together (Yang et al., 2010). Nevertheless, it has been recently disputed how an estimator should be adjusted under genetic architecture. Speed et al. (2012) suggested using a weighted genetic relationship matrix under different genetic architecture, which is often unknown. As demonstrated in large-scale empirical data studies (Lee et al., 2013), Speed's *ad-hoc* weighing method depends on the genetic architecture and does not often outperform plain weight methods upon comparison. As the genetic architecture, such as the relationship between variant frequency and variant effect, is often unknown, criteria should be established to justify the model used to estimate heritability.

For GWAS, as many samples are collected to study diseases, many studies eventually adopt a case-control design. Due to ascertainment in case-control studies, scale transformation is necessary. Without scale transformation, the heritability on the observed scale can be greater than 1, rendering the estimated heritability meaningless, as it is not representative of its heritability on the liability scale, which is more interpretable (Falconer, 1966) for disease data. An equation (Lee et al., 2011) that transforms heritability from the observed scale to the liability scale has been proposed (as the equation was indexed as the 23rd equation in Sang Hong Lee's paper, it is henceforth denoted as Hong23) and was investigated under the infinitesimal model, for which the number of casual loci is infinite. However, in practice, disease loci are reasonably limited for many diseases (Yang et al., 2011), which raises the question of whether or not Hong23 works well for mixed linear model estimates if the infinitesimal model does not hold.

All of the above concerns are related to the heritability estimated via variance component methods implemented thus far in mixed linear models. The Haseman–Elston (HE) regression is a prestigious method for estimating variance components (Haseman and Elston, 1972). The HE regression, a well-known tool for linkage studies that uses identity by descent (IBD) (Lynch and Walsh, 1998; Hill and Weir, 2011) scores, however, seems a rusty weapon in the genomics analysis armory of the GWAS era. This is because the HE regression relies on relatedness measured on IBD but not identity by state (IBS). Although IBS has been employed for linkage analysis, such as under affected-pedigree-member design (Lange, 1986b; Weeks and Lange, 1988; Bishop and Williamson, 1990), its performance is largely dependent on marker polymorphisms and may cause high false positives when *ad-hoc* weighting functions or incorrect frequencies are adopted. As an underrepresented concept of the linkage era, IBS is neither well-adapted to linkage studies nor employed in the original HE regression framework.

Taken together, the following questions remain.

1. Can the HE regression be applied to the IBS content such as for GWAS? If the answer is affirmative, what is the theoretical basis and the genetic interpretation in this new context?
2. An equilibrium has been established between the HE regression and the variance component method (Sham et al., 2002) for linkage studies. Does this equilibrium stand for high-dimensional data such as GWAS data and what are its assumptions?
3. If the IBS-based HE regression is applied to case-control studies, can it estimate heritability better than the mixed linear models?
4. Given the recent dispute regarding heritability estimation of complex traits, can HE regression provide further justification?

Recently, a new theory using like-standardized IBS has paved another route to assess genetic relatedness (Ritland, 1996; Powell et al., 2010; Yang et al., 2010) between unrelated individuals (conventional sense). The IBS score resembles the conventional IBD score (Powell et al., 2010), which raises the question of whether this IBS score can be used in the HE regression for unrelated individuals. In this study, by replacing the IBD scores with standardized IBS scores, we used the HE regression to conduct association studies for GWAS data. Assuming random mating, biallelic loci, and additive genetic effects only on the genetic architecture of quantitative trait loci (QTLs) underlying a complex trait, this report establishes the theoretical basis for using the HE regression for GWAS. Two generic scenarios were investigated, and their regression coefficients were derived and have genetically meaningful interpretations. In scenario I, the IBS score was assessed via a marker that was in linkage disequilibrium (LD) with a QTL. This enabled the HE regression to be a tool for single-marker GWAS. In scenario II, IBS score was assessed on multiple markers, each of which could be in LD with multiple QTLs. This allowed the HE regression to be used to estimate the variance component tagged by markers.

The second scenario has implications for estimation of heritability for complex trait using whole genome-wide markers together, similar to the mixed linear model (Yang et al., 2010; Lee et al., 2011). Using an analytical method that establishes the equivalence between the IBS-based HE regression and the mixed linear model, a simple criterion is proposed to justify the estimates in this study. A similar equivalence between the HE regression and the variance component analysis with the mixed linear model was determined in the context of linkage analysis (Sham and Purcell, 2001). In this study, their equivalence is established under the context of GWAS, and the conditions for equivalence are explored analytically as well as *in silico*. After extending the established HE regression into case-control scenarios, we demonstrated that Hong23 fits the estimate from the HE regression better than that from the mixed linear model.

Furthermore, as the IBS-based HE regression uses least squares, it is advantageous in its computational efficiency and is *N* (*N* is the sample size) times faster than the mixed linear model. In order to facilitate the application, the HE regression algorithm for GWAS data was implemented in Java software, GEnetic Analysis Repository (GEAR), which is freely available online.

As the first half of this report is focused on establishing the mathematical basis of the IBS-based HE regression, many mathematical symbols are introduced (Table 1). In the text below, the HE regression is the IBS-based HE regression unless explicitly noted otherwise.

**Table 1:** **Notation definitions**.

**Notation**	**Definition**
*p_k_* and *q_k_*	Allele frequencies of *A* and *a* at the *k^th^* locus. *A* is the reference allele.
*D*_*kl*_	Linkage disequilibrium of a pair of loci, *D*_*kl*_ = *f*_*a_k_a_l_*_ − *q_k_q_l_*, in which *f_a_k_a_l__* is the frequency of haplotype *a_k_a_l_*.
*r*_*kl*_ and *R*_*kl*_	*r*_*kl*_ = *p*(*a_l_*|*a_k_*) and *R*_*kl*_ = *p*(*A_l_*|*A_k_*), the conditional probabilities of the two coupling haplotypes, *a_k_a_l_* and *A_k_A_l_*.
ρ_*kl*_	$\rho_{kl}=\frac{D_{kl}}{\sqrt{p_{k}q_{k}p_{l}q_{l}}}$, the Pearson's correlation between a pair of biallelic loci, *k* and *l*.
ρ^2^_*M*_	The mean of the squared correlation between any marker pair, including the marker with itself. This can be estimated from the genotype data.
ρ^2^_*Q*_	The mean of the squared correlation between any a marker and a QTL.
Λ	The ratio between ρ^2^_*Q*_ and ρ^2^_*M*_. This indicates how markers tag causal variants.
*M*	The number of markers.
*M_e_*	The effective number of markers. See the text and Supplementary Note II for definition.
*x_i_*	*x_i_* = [*x*_*i*1_, *x*_*i*2_, *x*_*i*3_, …, *x*_*iM*_], genotype scores, a vector. It counts the reference allele number for each locus.
*g_k_*	The genotype set for the *k^th^* locus, such as *g_k_* = {*a_k_a_k_, A_k_a_k_, A_k_A_k_*}. Analogously, for a QTL, *g_k_* = { 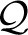 _*k*_ 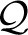 _*k*_, 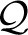 _*kq_k_, q_k_q_k_*_}.
*s_i_*	Standardized genotype scores for the *i*^*th*^ individual, a vector. $s_{i}=\left[\frac{x_{i1}-2p_{1}}{\sqrt{2p_{1}q_{1}}},\frac{x_{i2}-2p_{2}}{\sqrt{2p_{2}q_{2}}},\ldots ,\frac{x_{iM}-2p_{M}}{\sqrt{2p_{M}q_{M}}}\right]$.
*L*	The number of QTLs.
*N*	Sample size.
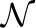	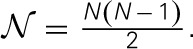 .
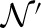	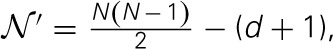 , in which *d* is the number of parameters in the HE regression.
*y_i_*	The phenotype of the *i^th^* individual.
*Y*_ij_	The square of the phenotype difference between the *i^th^* and the *j^th^* individuals.
Ω_*ij*_	The genetic relatedness between the *i^th^* and the *j^th^* individuals. See the text for definition.
β_*l*_	The additive effect of the *l^th^* QTL.
σ^2^_*A*_	Total additive variance.
*h*^2^	Narrow-sense heritability.
σ_*l*_	The square-root of the additive variance of the *l^th^* QTL, $\sigma_{l}=\sqrt{2p_{l}q_{l}}\beta_{l}$.
Hong23	Expressed as $h_{l}^{2}=h_{o}^{2}\frac{K(1-K)}{z^{2}}\frac{K(1-K)}{P(1-P)}$ is the heritability on the liability scale, *h*^2^_*o*_ is the heritability on the observed scale directly estimated based on the case-control data, *K* is the disease prevalence, *P* is the proportion of cases in the data, and *z* is the height of the standard normal distribution in which the prevalence is located (Lee et al., 2011).
Subscript	Subscripts *i* and *j* are used to indicate individuals, and *k* and *l* are used to indicate loci, which can be either markers or QTLs.

## Theory of the IBS-based He regression

For an individual, the phenotype is denoted as *y*_*i*_, which follows the normal distribution of *N*(μ_*y*_, σ^2^_*y*_), and the genotype is *x*_*i*_ = [*x*_*i*1_, *x*_*i*2_, …, *x*_*iM*_], in which *M* is the number of markers. For the *i*^*th*^ individual, the genotype at the *k*^*th*^ locus is *x*_*ik*_, which counts the reference alleles at the *k*^*th*^ locus. The reference allele is denoted as *A*_*k*_ and the alternative is *a*_*k*_. The frequency of *A*_*k*_ is *p*_*k*_ and the frequency of *a*_*k*_ is *q*_*k*_. *g*_*k*_ is the set of possible genotypes, say {*a_*k*_a_*k*_, *A*_*k*_a_*k*_, A_*k*_A_*k*_*}, at the *k*^*th*^ locus. Consequently, *a*_*k*_*a*_*k*_, *A*_*k*_*a*_*k*_, and *A*_*k*_*A*_*k*_ are coded as 0, 1, and 2, respectively. After standardization, *x*_*i*_ is expressed as $s_{i}=\left[\frac{x_{i1}-2p_{1}}{\sqrt{2p_{1}q_{1}}},\frac{x_{i2}-2p_{2}}{\sqrt{2p_{2}q_{2}}},\ldots ,\frac{x_{iM}-2p_{M}}{\sqrt{2p_{M}q_{M}}}\right]$. For *x*_*ik*_, given a genotype of *a*_*k*_*a*_*k*_, *A*_*k*_*a*_*k*_, and *A*_*k*_*A*_*k*_, their standardized scores are $\frac{-2p_{k}}{\sqrt{2p_{k}q_{k}}},\frac{q_{k}-p_{k}}{\sqrt{2p_{k}q_{k}}}$, and $\frac{2q_{k}}{\sqrt{2p_{k}q_{k}}}$, respectively. The additive effect of the *l*^*th*^ QTL is denoted as β_*l*_. Throughout the study, we assume a polygenic model with *L* QTLs.

### The IBS-based he regression

Haseman and Elston (1972) proposed a linear model, *Y*_*ij*_ = μ + *b*π_*ij*_ + *e*_*ij*_, for detecting linkage between a marker and a QTL in a full-sib design. *Y*_*ij*_ represents the squared difference between a pair of full sibs, and π_*ij*_ is the proportion of IBD at an observed marker locus; μ is the intercept of the regression, *b* is the regression coefficient, and *e*_*ij*_ is the residual. The mathematical expectation of the regression coefficient is *b* = −2(1 − 2*c*)^2^ σ^2^_*A*_, in which *c* is the recombination fraction between the marker locus and the QTL, and σ^2^_*A*_ is the additive genetic variance of the QTL.

Now consider a sample consisting of *N* unrelated individuals. If the phenotype for the *i*^*th*^ individual is *y*_*i*_, we can modify the HE original regression as below

(1) $$Y_{ij}=\mu +b\Omega_{ij}+e_{ij}$$

in which *Y*_*ij*_ = (*y*_*i*_ − *y*_*j*_)^2^ represents the squared difference, Ω_*ij*_ is the measure of the genetic relatedness of a pair of individuals, and *e*_*ij*_ is the residual. Given *N* unrelated individuals, there are 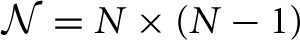 such individual pairs. Ω_*ij*_ is the similarity score between a pair of individuals based on the IBS, as recently proposed (Powell et al., 2010; Yang et al., 2010).

For the linear model in Equation (1), the expectation of the regression coefficient is $E(b)=\frac{cov(Y_{ij},\Omega_{ij})}{var(\Omega_{ij})}$. *var*(Ω_*ij*_) is the variance of the genetic relatedness. *cov*(*Y*_*ij*_, Ω_*ij*_) = *E*(Ω_*ij*_*Y*_*ij*_) − *E*(Ω_*ij*_)*E*(*Y*_*ij*_) = *E*(Ω_*ij*_*Y*_*ij*_) because *E*(Ω_*ij*_) = 0 [see the definition for Ω_*ij*_ in section The Derivation of *var*(Ω_*ij*_) and Effective Number of Markers (*M*_*e*_)], and $E\left(\Omega_{ij}Y_{ij}\right)=\Sigma_{k\;=\;1}^{M}\Sigma_{x_{ik}\in g_{k}}\Sigma_{x_{jk}\in g_{k}}s_{ik}s_{jk}{\left[E\left(y_{i}|x_{ik}\right)-E\left(y_{j}|x_{jk}\right)\right]}^{2}p\left(x_{ik}\right)p(x_{jk})$ is the mathematical expectation of the joint distribution for Ω_*ij*_ and *Y*_*ij*_. In order to derive *var*(Ω_*ij*_) and *cov*(*Y*_*ij*_, Ω_*ij*_), we need to introduce the haplotype distribution of a biallelic loci pair (section Haplotypes of a Biallelic Loci Pair). When the haplotype is constructed on a pair of markers, it leads to the derivation of *var*(Ω_*ij*_) [section The Derivation of *var*(Ω_*ij*_) and Effective Number of Markers (*M*_*e*_)]; when the haplotype is constructed for a marker and a QTL, it leads to *E*(*y*_*i*_|*x*_*ik*_), the conditional expectation of the phenotype based on a marker [section The Derivation of *E*(*y_i_*|*x_ik_*)].

### Derivations of *var*(Ω_*ij*_) and *E*(*y_i_|x_ik_*)

#### Haplotypes of a biallelic loci pair

For a pair of biallelic loci, there are four haplotype phases, and their conditional probabilities are as summarized in Table S1. *r_kl_* = *p*(*a_l_*|*a_k_*) and *R_kl_* = *p*(*A_l_*|*A_k_*) are defined as the conditional probabilities of the haplotypes in the coupling phases, such as *a_k_a_l_* and *A_k_A_l_*, respectively; 1 − *r_kl_* and 1 − *R_kl_* represent the conditional probabilities of the alleles in their repulsion phases, such as *a_k_A_l_* and *A_k_a_l_*, respectively. *D_kl_* = *f_A_k_A_l__* − *p_k_p_l_*, in which *f_A_k_A_l__* is the frequency of the haplotype *A_k_A_l_*; *D_kl_* is the covariance between the loci, quantifying the LD between them.

The correlation of a pair of biallelic loci can be expressed as a 2 × 2 correlation

(2) $$\rho_{kl}=\frac{D_{kl}}{\sqrt{p_{k}q_{k}p_{l}q_{l}}}$$

ρ^2^_*kl*_ is often used to parameterize the LD of a loci pair (Hill and Robertson, 1968). For more LD parameterization, please refer to Devlin and Risch (1995) and Wray (2005).

Once the conditional probabilities of the haplotypes are defined, it is straightforward to obtain the joint probabilities of the genotypes for a pair of loci. For example, under random mating, the probability of the genotype *A_k_A_k_A_l_A_l_* is *p(A_k_A_k_A_l_A_l_)* = *p(A_k_A_k_|A_l_A_l_)p(A_k_A_k_)* = *p(A_l_|A_k_)p(A_k_) p(A_l_|A_k_)p(A_k_)* = *p*^2^*_k_R*^2^*_kl_*. Analogously, this leads to the joint probabilities of the other eight two-locus genotypes (See Table 2).

**Table 2:** **The joint distribution of two loci**.

		**The *k*^th^ locus**		
		***a_k_a_k_***	***A_k_a_k_***	***A_k_A_k_***
The *l*^*th*^ locus	*a_l_a_l_*	*q*^2^_*k*_*r*^2^_*kl*_	2*p_k_q_k_r_kl_(1 − *R*_kl_*)	*p*^2^_*k*_(1 − *R_kl_*)^2^
	*A_l_a_l_*	2*q*^2^_*k*_*r_kl_*(1 − *r_kl_*)	2*p_k_q_k_*[*r_kl_R_kl_* + (1 − *r_kl_*) (1 − *R_kl_*)]	2*p*^2^*_k_R_kl_*(1 − *R_kl_*)
	*A_l_A_l_*	*q*^2^_*k*_(1 − *r_kl_*)^2^	2*p_k_q_k_R_kl_*(1 − *r_kl_*)	*p*^2^_*k*_*R*^2^_*kl*_
Marginal probability		*q*^2^_*k*_	2*p_k_q_k_*	*p*^2^_*k*_

*Each cell lists the joint probability of a genotype pair at the *k*^th^ and the l^th^ locus, respectively*.

*r_kl_ and R_kl_, as defined in Table S1, represent the conditional probabilities of the haplotypes a_k_a_l_ and A_k_A_l_, respectively*.

#### The derivation of *var*(Ω_*ij*_) and effective number of markers (*M_e_*)

For a sample consisting of unrelated individuals, their pairwise genetic relationships, say additive genetic relationships, can be estimated with genetic markers, such as SNP markers (Powell et al., 2010; Yang et al., 2010). The genetic relatedness Ω_*ij*_ between the *i*^*th*^ individual and the *j*^*th*^ individual is measured by the dot product of their standardized genotypes and then divided by the number of markers.

(3) $$\Omega_{ij}=\frac{s_{i}.s_{j}}{M}=\frac{1}{M}\Sigma_{k\;=\;1}^{M}\frac{\left(x_{ik}-2p_{k}\right)}{\sqrt{2p_{k}q_{k}}}\frac{\left(x_{jk}-2p_{k}\right)}{\sqrt{2p_{k}q_{k}}}$$

The possible relatedness scores of a pair of individuals are summarized in Table 3A, totaling nine products. After combining the same score values, there are seven unique terms as in Table 3B. It is easy to derive that *E*(Ω_*ij*_) = 0 and $var\left(\Omega_{ij}\text{​}\right)=\frac{1}{M^{2}}\Sigma_{k\;=\;1}^{M}\Sigma_{l\;=\;1}^{M}cov\left(\Omega_{ijk},\Omega_{ijl}\right)$, in which *cov*(Ω_*ijk*_, Ω_*ijl*_) = *E*(Ω_*ijk*_Ω_*ijl*_) − *E*(Ω_*ijk*_)*E*(Ω_*ijk*_) = *E*(Ω_*ijk*_Ω_*ijl*_) because *E*(Ω_*ij*._) = 0.

**Table 3A:** **The joint distribution of the genetic relatedness between individuals *i* and *j***.

**Individual *i***			**Individual *j***			**Relatedness for individuals *i* and *j***	
**Genotype**	***s_*ik*_***	**Frequency**	**Genotype**	***s_k_***	**Frequency**	**Ω_*ijk*_**	**Frequency**
*a_k_a_k_*	$\frac{-2p_{k}}{\sqrt{2p_{k}q_{k}}}$	*q*^2^_*k*_	*a_k_a_k_*	$\frac{-2p_{k}}{\sqrt{2p_{k}q_{k}}}$	*q*^2^_*k*_	$\frac{4p_{k}^{2}}{2p_{k}q_{k}}$	*q*^4^_*k*_
			*A_k_a_k_*	$\frac{q_{k}-p_{k}}{\sqrt{2p_{k}q_{k}}}$	2*p_k_q_k_*	$\frac{-2p_{k}(q_{k}-p_{k})}{2p_{k}q_{k}}$	2*p_k_q*^3^_*k*_
			*A_k_A_k_*	$\frac{2q_{k}}{\sqrt{2p_{k}q_{k}}}$	*p*^2^_*k*_	$\frac{-4p_{k}q_{k}}{2p_{k}q_{k}}$	*p*^2^_k_q^2^_*k*_
*A_k_a_k_*	$\frac{q_{k}-p_{k}}{\sqrt{2p_{k}q_{k}}}$	2*p_k_q_k_*	*a_k_a_k_*	$\frac{-2p_{k}}{\sqrt{2p_{k}q_{k}}}$	*q*^2^_*k*_	$\frac{-2p_{k}(q_{k}-p_{k})}{2pq}$	2*p_k_q*^3^_*k*_
			*A_k_a_k_*	$\frac{q_{k}-p_{k}}{\sqrt{2p_{k}q_{k}}}$	2*p_k_q_k_D*	$\frac{{\left(q_{k}-p_{k}\right)}^{2}}{2p_{k}q_{k}}$	4*p*^2^*_k_q*^2^*_k_*
			*A_k_A_k_*	$\frac{2q_{k}}{\sqrt{2p_{k}q_{k}}}$	*p*^2^_*k*_	$\frac{2q(q_{k}-p_{k})}{2p_{k}q_{k}}$	2*p*^3^*_k_q_k_*
*A_k_A_k_*	$\frac{2q_{k}}{\sqrt{2p_{k}q_{k}}}$	*p*^2^_*k*_	*a_k_a_k_*	$\frac{-2p_{k}}{\sqrt{2p_{k}q_{k}}}$	*q*^2^_*k*_	$\frac{-4p_{k}q_{k}}{2p_{k}q_{k}}$	*p*^2^_k_q^2^_k_
			*A_k_a_k_*	$\frac{q_{k}-p_{k}}{\sqrt{2p_{k}q_{k}}}$	2*p_k_q_k_*	$\frac{2q(q_{k}-p_{k})}{2p_{k}q_{k}}$	2*p*^3^*_k_q_k_*
			*A_k_A_k_*	$\frac{2q_{k}}{\sqrt{2p_{k}q_{k}}}$	*p*^2^_*k*_	$\frac{4q_{k}^{2}}{2p_{k}q_{k}}$	*p*^4^_*k*_

*As A was set as the reference allele, with a frequency of p, aa, Aa, and AA were coded as 0, 1, and 2, respectively*.

**Table 3B:** **A reorganization of Table 3A to illustrate the relatedness joint distribution of a pair of individuals**.

Ω_*ijk*_	$\frac{4p_{k}^{2}}{2p_{k}q_{k}}$	$\frac{-2p_{k}(q_{k}-p_{k})}{2p_{k}q_{k}}$	$\frac{-4p_{k}q_{k}}{2p_{k}q_{k}}$	$\frac{{\left(q_{k}-p_{k}\right)}^{2}}{2p_{k}q_{k}}$	$\frac{2q_{k}(q_{k}-p_{k})}{2p_{k}q_{k}}$	$\frac{4q_{k}^{2}}{2p_{k}q_{k}}$
Frequency	*q*^4^_*k*_	4*p*_*k*_*q*^3^_*k*_	2*p*^2^_*k*_*q*^2^_*k*_	4*p*^2^_*k*_*q*^2^_*k*_	4*p*^3^_*k*_*q*_*k*_	*p*^4^_*k*_

*Ω_ijk_ represents the product of a standardized genotype pair for individual i and individual j on the k^th^ locus*.

Ω_*ij*_ is informative in revealing hidden relatedness. For example, for the duplicated individual in the sample, *E*(Ω_*ij*_) = 1; for first-degree relatives, *E*(Ω_*ij*_) = 0.5; for second-degree relatives, *E*(Ω_*ij*_) = 0.25. Consequently, it can control the entry of samples that are under the expected cutoff for relatedness.

After some additional algebra (see Supplementary Note I), we arrived at the following equation.

(4) $$cov\left(\Omega_{ijk},\Omega_{ijl}\right)=\rho_{kl}^{2}$$

When the *k*^*th*^ locus and the *l*^*th*^ locus are in linkage equilibrium, *cov*(Ω_*ijk*_, Ω_*ijl*_) = 0; when the *k*^*th*^ locus and the *l*^*th*^ locus are at the same locus, *cov*(Ω_*ijk*_, Ω_*ijl*_) = 1.

(5) $$var\left(\Omega_{ij}\right)=\frac{1}{M^{2}}\Sigma_{k\;=\;1}^{M}\Sigma_{l\;=\;1}^{M}\rho_{kl}^{2}=\frac{1}{M}+\frac{1}{M^{2}}\Sigma_{k\;=\;1}^{M}\Sigma_{l\;\neq \;k}^{M}\rho_{kl}^{2}$$

The distribution of ρ^2^_*kl*_ varies with *p_k_* and *p_l_* (Wray, 2005). We can also interpret *var*(Ω_*ij*_) as the mean of the squared Pearson's correlation between the markers along the genome, denoted as ρ^2^_*M*_.

For simplicity of the following derivation, the concept of an effective number of markers, *M_e_*, is introduced here. Intuitively, as markers are often in linkage disequilibrium, the real number of “independent” markers is smaller than the total number of the markers genotyped. This concept was previously introduced under the context of risk prediction (Purcell et al., 2009), and *M_e_* was evaluated using Monte Carlo simulation. As indicated in Supplementary Note II, 1/*var*(Ω_*ij*_) is the mathematical expectation of the effective number of markers evaluated under the simulation method (Purcell et al., 2009). For example, for 100 equifrequent biallelic loci, if the correlation for each pair of consecutive markers is 0, 0.25, 0.5, and 0.75, the effective number of markers is approximately 100, 90, 61, and 29, respectively. Real GWAS data are often at a magnitude of 10^4^ (Vinkhuyzen et al., 2013).

#### The derivation of *E*(*y_i_*|*x_ik_*)

The expected phenotype of *y_i_* given genotype *x_ik_* depends on the QTL genotype, say the *l*^*th*^ locus, in LD with *x_ik_*. Assuming a biallelic QTL in LD with the marker, the conditional expectation of the marker is *E*(*y_i_|x_ik_* = *A_k_A_k_*) = Σ*_x_ilgl__s_il_p(x_il_|x_ik_* = *A_k_A_k_*), in which *g_l_* = {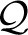
_*l*_ 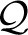
_*l*_, 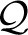
_*l*_*q_l_*, *q_l_q_l_*}, and *E*(*y_i_*|*x_ik_* = *A_k_A_k_*) = β_*l*_ × *R_kl_*^2^ + 0 × 2*R_kl_* (1 − *R_kl_*) − β_*l*_ × (1 − *R_kl_*)^2^ = (2*R_kl_* − 1)β_*l*_. Analogously, we can derive the expected values of *E*(*y_i_|x_ik_* = *A_k_a_k_*) = (*R_kl_* − *r_kl_*)β_*l*_ and *E*(*y_i_|x_ik_* = *a_k_a_k_*) = (1 − 2*r_kl_*)β_*l*_ (See Table 4). Once *E*(*y_i_|x_ik_*) is defined, the distribution of *E*(*Y_*ij*_|x_ik_, x_jk_*) can be tabulated as in Table 5.

**Table 4:** **The expected phenotype conditional to one's genotype on the observed marker**.

**Marker genotype**	**QTL genotype**	**QTL conditional probability**	***E(y_i_|x_ik_)***
*a_k_a_k_*	*q_l_q_l_*	*r*^2^_*kl*_	(1 − 2*r_kl_*)β_*l*_
	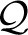 *_l_q_l_*	*r*_*kl*_(1 − *r_kl_*)	
	*q_l_* 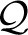 _*l*_	*r_kl_*(1 − *r_kl_*)	
	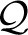 _*l*_ 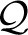 _*l*_	(1 − *r_kl_*)^2^	
*A_k_a_k_*	*q_l_q_l_*	*r*_*kl*_(1 − *R_kl_*)	(*R_kl_* − *r_kl_*)β_*l*_
	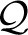 *_l_q_l_*	(1 − *r_kl_*)(1 − *R_kl_*)	
	*q_l_* 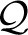 _*l*_	*r_kl_R_kl_*	
	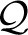 _*l*_ 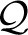 _*l*_	(1 − *r_kl_*)	
*A_k_A_k_*	*q_l_q_l_*	(1 − *R_kl_*)^2^	(2*R_kl_* − 1)β_*l*_
	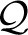 *_l_q_l_*	*R*_*kl*_(1 − *R_kl_*)	
	*q_l_* 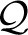 _*l*_	*R_kl_*(1 − *R_kl_*)	
	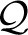 _*l*_ 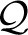 _*l*_	*R*^2^_*kl*_	

*It is assumed that the k^th^ locus is the observed marker and the l^th^ locus is the QTL*.

*r_kl_ and R_kl_ are the conditional probabilities of the coupling phases of the haplotypes as defined in Table S1*.

**Table 5:**
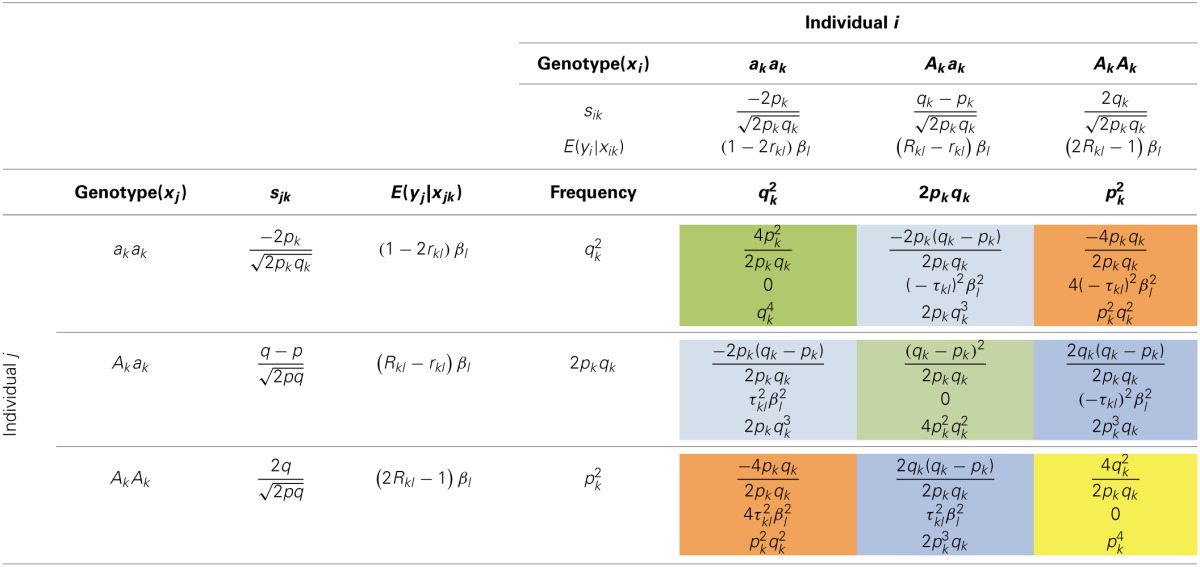
**The joint distribution of *E*(Ω_*ij*_) and *E*(*Y_*ij*_*|*x_i_, x_j_*) for one marker and one QTL**.

*s_.k_ represents the standardized genotypes of the k^th^ locus*.

*For the nine cells, the symmetrical cells are highlighted in same color. In each highlighted cell, three terms from the top to the bottom are Ω_*ij*_ = s_ik_s_jk_, E(Y_*ij*_|x_ik_, x_jk_) = [E(y_i_|x_ik_) − E(y_j_|x_jk_)]^2^ and their frequencies*.

*τ_kl_ = 1 − r_kl_ − R_kl_*.

### Deriving the mathematical expectation of the regression coefficient

In this section, we investigated two scenarios to derive the expected value of the regression coefficient for Equation (1). In scenario I, genetic similarity is estimated at a single marker, which is in LD with one or more QTLs. In scenario II, genetic similarity is estimated based on *M* markers, each of which can be in LD with *L* QTLs.

#### Scenario I: one marker and one QTL

Under the scenario of one marker, say the *k*^*th*^ marker, and one QTL, say the *l*^*th*^ QTL, since *var*(Ω_*ijk*_) = 1, *E*(*b*) = *E*(Ω*_ij_Y_ij_*), which is *E*(Ω*_ij_Y_ij_*) = Σ*_x_ik__*Σ*_x_jk__s_ik_s_jk_*[*E(y_i_|x_ik_)* − *E(y_j_|x_jk_)*]^2^*p*(*x_ik_*)*p(x_jk_)*. Consequently, we can derive the regression coefficient as

$$\begin{array}{l} E\left(b\right)=E\left(\Omega_{ij}Y_{ij}\right)=\Sigma_{x_{ik}}\Sigma_{x_{jk}}s_{ik}s_{jk}[E\left(y_{i}|x_{ik}\right)\\ {\;-E\left(y_{j}|x_{jk}\right)]}^{2}p\left(x_{ik}\right)p(x_{jk})=\frac{-2p_{k}\left(q_{k}-p_{k}\right)}{2p_{k}q_{k}}\tau_{kl}^{2}\beta_{l}^{2}2p_{k}q_{k}^{3}\\ \;+\;\frac{-2p_{k}\left(q_{k}-p_{k}\right)}{2p_{k}q_{k}}{\left(-\tau_{kl}\right)}^{2}\beta_{l}^{2}2p_{k}q_{k}^{3}+\frac{-4p_{k}q_{k}}{2p_{k}q_{k}}4\tau_{kl}^{2}\beta_{l}^{2}p_{k}^{2}q_{k}^{2}\\ \;+\;\frac{-4p_{k}q_{k}}{2p_{k}q_{k}}4{\left(-\tau_{kl}\right)}^{2}\beta_{l}^{2}p_{k}^{2}q_{k}^{2}+\frac{2q_{k}\left(q_{k}-p_{k}\right)}{2p_{k}q_{k}}\tau_{kl}^{2}\beta_{l}^{2}2p_{k}^{3}q_{k}\\ \;+\frac{2q_{k}\left(q_{k}-p_{k}\right)}{2p_{k}q_{k}}{\left(-\tau_{kl}\right)}^{2}\beta_{l}^{2}2p_{k}^{3}q_{k} \end{array}$$

and

(6) $$E(b)=-4\tau_{kl}^{2}p_{k}q_{k}\beta_{l}^{2}$$

in which τ_*kl*_ = 1 − *r_kl_* − *R_kl_*.

When the QTL overlaps with the marker, or the correlation between the QTL and the marker is 1, *E*(*b*) = −4*p_k_q_k_* β^2^_*l*_ because *r*_*kl*_ = *R_kl_* = 1. When the QTL is in linkage equilibrium with the marker, *r*_*kl*_ = *q_l_* and *R*_*kl*_ = *p_l_*, 1 − *r_kl_* − *R_kl_* = 0, and consequently *E*(*b*) = 0.

According to Table S1, $r_{kl}+R_{kl}=\left(q_{l}+\frac{D_{kl}}{q_{k}}\right)+\left(p_{l}+\frac{D_{kl}}{p_{k}}\right)=1+\frac{D_{kl}}{p_{k}q_{k}}$. Consequently, the expression of Equation (6) can be rearranged as $E(b)=-4{\left(\frac{D_{kl}}{p_{k}q_{k}}\right)}^{2}p_{k}q_{k}\beta_{l}^{2}$. The correlation of a pair of biallelic loci $\rho_{kl}=\frac{D_{kl}}{\sqrt{p_{k}q_{k}p_{l}q_{l}}}$ [Equation (2)], and consequently *E(b)* = − 2ρ^2^_*kl*_σ^2^_*l*_, in which $\sigma_{l}=\sqrt{2p_{l}q_{l}}\beta_{l}$. Alternatively, we can write

(7) $$E(b)=-2\rho_{kl}^{2}\sigma_{l}^{2}.$$

In the GWAS context, squared LD (Pearson's correlation) is *in lieu* of the recombination fraction for linkage. The mathematical expectation of the regression coefficient resembles the one in the original HE regression. However, it should be noted that here the interpretation of the regression coefficient is based on linkage disequilibrium and association, whereas the original interpretation is based on linkage between the marker and the QTL.

When multiple QTLs are in LD with the marker, the conditional expectation for *y*_*i*_ given *x*_*ik*_ is $E\left(y_{i}|x_{ik}=A_{k}A_{k}\right)=\Sigma_{l}^{L}(2R_{kl}-1)\beta_{l},\;E\left(y_{i}|x_{ik}=A_{k}a_{k}\right)=\Sigma_{l}^{L}(R_{kl}-r_{kl})\beta_{l}$, and $E\left(y_{i}|x_{ik}=a_{k}a_{k}\right)=\Sigma_{l}^{L}(1-2r_{kl})\beta_{l}$, respectively. The joint distribution of Ω_*ij*_ and *Y_ij_* is as summarized in Table S2, which resembles Table 5. Still using *E*(Ω*_ij_Y_ij_*) = Σ*_x_ik__Σ_x_jk__s_ik_s_jk_[E(y_i_|x_ik_)* − *E(y_j_|x_jk_)*]^2^*p(x_ik_)p(x_jk_)*, the regression coefficient can be derived as below.

(8) $$\begin{array}{l} E(b)=\frac{-2p_{k}\left(q_{k}-p_{k}\right)}{2p_{k}q_{k}}{\left[\Sigma_{l=1}^{L}\tau_{kl}\beta_{l}\right]}^{2}2p_{k}q_{k}^{3}\\ \;+\frac{-4p_{k}q_{k}}{2p_{k}q_{k}}4{\left[\Sigma_{l=1}^{L}\tau_{kl}\beta_{l}\right]}^{2}p_{k}^{2}q_{k}^{2}\\ \;+\frac{2q_{k}\left(q_{k}-p_{k}\right)}{2p_{k}q_{k}}{\left[\Sigma_{l=1}^{L}\tau_{kl}\beta_{l}\right]}^{2}2p_{k}^{3}q_{k}\\ \;+\frac{-2p_{k}\left(q_{k}-p_{k}\right)}{2p_{k}q_{k}}{\left[\Sigma_{l=1}^{L}-\tau_{kl}\beta_{l}\right]}^{2}2p_{k}q_{k}^{3}\\ \;+\frac{-4p_{k}q_{k}}{2p_{k}q_{k}}4\left[\Sigma_{l=1}^{L}-\tau_{kl}\beta_{l}\right]p_{k}^{2}q_{k}^{2}\\ \;+\frac{2q_{k}\left(q_{k}-p_{k}\right)}{2p_{k}q_{k}}{\left[\Sigma_{l=1}^{L}-\tau_{kl}\beta_{l}\right]}^{2}2p_{k}^{3}q_{k}\\ \;=-4p_{k}q_{k}{\left[\Sigma_{l=1}^{L}\tau_{kl}\beta_{l}\right]}^{2} \end{array}$$

Equation (8) can be rearranged as

(9) $$E\left(b\right)=-2\Sigma_{l_{1}=1}^{L}\Sigma_{l_{2}=1}^{L}\rho_{kl_{1}}\rho_{kl_{2}}\sigma_{l_{1}}\sigma_{l_{2}}.$$

It is easy to see that when *L* = 1, Equation (9) can be simplified to Equation (7).

#### Scenario II: multiple markers and multiple QTLs

When the genetic relatedness matrix is constructed with *M* markers, each of which may be in LD with *L* QTLs, the HE regression becomes *Y*_*ij*_ = *a* + *b*Ω_*ij*_, in which $\Omega_{ij}=\frac{1}{M}\Sigma_{k}^{M}s_{ik}s_{jk}$. For convenience, Ω_*ijk*_ denotes the relatedness fraction constructed with the *k*^*th*^ marker between the *i*^*th*^ and the *j*^*th*^ individuals. According to the definition of the regression coefficient, $b=\frac{Cov(\Omega_{ij},Y_{ij})}{var(\Omega_{ij})}$.

$$\begin{array}{l} cov\left(\Omega_{ij},Y_{ij}\right)=\frac{1}{M}cov\left(\sum_{k=1}^{M}\Omega_{ijk},Y_{ij}\right)=\frac{1}{M}\sum_{k=1}^{M}cov\left(\Omega_{ijk},Y_{ij}\right)\\ \;=\frac{1}{M}\sum_{k=1}^{M}-4p_{k}q_{k}{\left[\Sigma_{l=1}^{L}\tau_{kl}\beta_{l}\right]}^{2} \end{array}$$

$var\left(\Omega_{ij}\right)=\Sigma_{k=1}^{M}\Sigma_{l=1}^{M}cov\left(\Omega_{ijk},X_{ijl}\right)/M^{2}$, in which *cov*(Ω*_ijk_, X_ijl_*) = ρ*_kl_*^2^, as expressed in Equation (4).

(10) $$E(b)=\left\{\frac{\Sigma_{k=1}^{M}-4p_{k}q_{k}{\left[\Sigma_{l=1}^{L}\tau_{kl}\beta_{l}\right]}^{2}}{M}\right\}/\left\{\frac{\Sigma_{k=1}^{M}\Sigma_{l=1}^{M}\rho_{kl}^{2}}{M^{2}}\right\}$$

After rearrangement

(11) $$E\left(b\right)=-2\sigma_{A}^{2}\Lambda +\Delta$$

in which $\sigma_{A}^{2}=\Sigma_{l}^{L}2p_{l}q_{l}\beta_{l}^{2}$, 
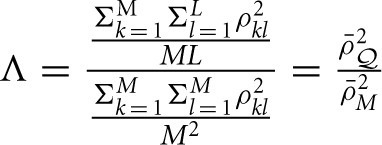
 and $\Delta =-2\Sigma_{k=1}^{M}(\text{​}\Sigma_{l_{1}=1}^{L}\Sigma_{l_{2}\neq l_{1}}^{L}2\rho_{kl_{1}}\rho_{kl_{2}}\sqrt{p_{l_{1}}q_{l_{1}}p_{l_{2}}q_{l_{2}}}\beta_{l_{1}}\beta_{l_{2}}\text{​})/M$, summarizing the between-locus variance. 
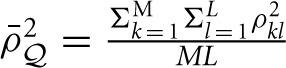
 is the average squared LD between a marker and a QTL across the genome, and ${\bar{\rho }}_{M}^{2}=\frac{\Sigma_{k=1}^{M}\Sigma_{l=1}^{M}\rho_{kl}^{2}}{M^{2}}$ is the averaged LD between every pair of markers, including the LD between each marker to itself. The interpretation of Equation (11) will be clear in Simulation III and Simulation IV.

If the phenotype is standardized, heritability equals the additive variance component. It is straightforward to obtain an estimate of the heritability for a single QTL, as in scenario I, or all QTLs, as in scenario II (See Supplementary Note IV)

(12) $$E\left(-\frac{b}{2}\right)=h^{2}\Lambda$$

### The sampling variance of the regression coefficient

The sum of square error (SSE) is

$$SSE=var\left(Y_{ij}\right)-{\hat{b}}^{2}var\left(\Omega_{ij}\right)$$

*var*(*Y_ij_*) = 8σ^4^_*y*_ (Supplementary Note III), and ${\hat{b}}^{2}var\left(\Omega_{ij}\right)=4\sigma_{A}^{4}\Lambda^{2}var\left(\Omega_{\text{ij}}\right)=4\sigma_{A}^{4}\frac{{\bar{\rho }}_{Q}^{4}}{{\bar{\rho }}_{M}^{2}}$

$$SSE=8\sigma_{y}^{4}-4\sigma_{A}^{4}.$$

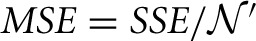
, in which 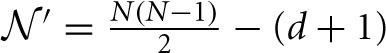 and *d* is the number of the regression coefficient (here *d* = 1).


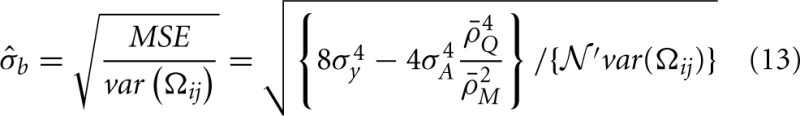


For scenario I, as only one marker is used, *var*(Ω_*ij*_) = 1 and ρ^2^_*M*_ = 1.


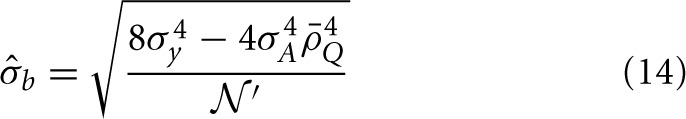


Given the current GWAS data, which incorporates thousands of individuals and often up to one million markers, it is reasonable to assume 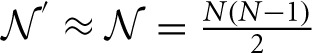 and 8*M*_*e*_ » 4σ^4^_*A*_ Λ^2^.

(15) $${\hat{\sigma }}_{b}\approx \sqrt{\frac{16M_{e}}{N(N-1)}}\approx \frac{4}{N}\sqrt{M_{e}}$$

For real GWAS data with about one million markers, $M_{e}=\frac{1}{var\left(\Omega_{ij}\right)}=\frac{1}{{\bar{\rho }}_{M}^{2}}$ ranges from 30,000 to 50,000 markers due to the strong LD pattern (Vinkhuyzen et al., 2013).

When the phenotype is standardized, the sampling variance of the regression coefficient is half of the additive variance component.

(16) $${\hat{\sigma }}_{h^{2}}=\frac{1}{2}{\hat{\sigma }}_{b}\approx \frac{2}{N}\sqrt{M_{e}}$$

### The mathematical expectation of the He regression intercept

The expectation of the intercept is *E*(*Y_ij_*) = *E*[(*y_i_* − *y_j_*)^2^] = *E*(*y_i_*^2^) + *E*(*y_j_*^2^) − 2*E*(*y_i_y_j_*). *E*(*y_i_*^2^) = *var*(*y_i_*) − *E*(*y_i_*)^2^, *E*(*y_j_*^2^) = *var*(*y_j_*) − *E*(*y_j_*)^2^, *E*(*y_i_y_j_*) = *cov*(*y_i_, y_j_*) + *E*(*y_i_*)*E*(*y_j_*). As the individuals are not related to each other, assuming no common environment, *cov*(*y_i_, y_j_*) = 0. So, *E*(*Y_ij_*) = *var*(*y_i_*) + *var*(*y_j_*) = 2σ^2^_*A*_ + 2σ^2^_*e*_, twice the phenotypic variance. The negative ratio between the regression coefficient and the intercept provides an estimate of the heritability if the phenotype is not standardized.

The derived regression coefficients and their sampling variance at the completion of the derivation are summarized in Table 6.

**Table 6:** **Summary of the derivations**.

**Scenario**	***E*(*b*)**		**σ_*b*_**
	**In genetic parameters**	**In statistical parameters**	
One marker and one QTL	−4τ^2^*_kl_p_k_q_k_*β^2^_*l*_	−2ρ^2^_*kl*_σ^2^_*l*_	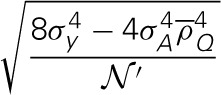
One marker and multiple QTLs	$-4p_{k}q_{k}{\left[\Sigma_{l=1}^{L}\tau_{kl}\beta_{l}\right]}^{2}$	$-2\Sigma_{l_{1}=1}^{L}\Sigma_{l_{2}=1}^{L}\rho_{kl_{1}}\rho_{kl_{2}}\sigma_{l_{1}}\sigma_{l_{2}}$	As above
Multiple markers and multiple QTLs	$\left\{\frac{\Sigma_{k=1}^{M}-4p_{k}q_{k}{\left[\Sigma_{l=1}^{L}\tau_{kl}\beta_{l}\right]}^{2}}{M}\right\}/\left\{\frac{\Sigma_{k=1}^{M}\Sigma_{l=1}^{M}\rho_{kl}^{2}}{M^{2}}\right\}$	−2σ^2^_*A*_Λ if QTLs are randomly allocated along the genome.	$\approx \frac{4}{N}\sqrt{M_{e}}$

*For *E*(b), the first expression is derived from the conditional probability and the second expression is for statistical neatness*.

*When the phenotype is standardized, h^2^ = −0.5b and σ_h^2^_ = 0.5σ_b_*.

### The additive variance component structure of a quantitative trait without ascertainment

The additive variance of a trait is defined as $\sigma_{A}^{2}=\Sigma_{l=1}^{L}2p_{l}q_{l}\beta_{l}^{2}+\Sigma_{l_{1}=1}^{L}\Sigma_{l_{2}\neq l_{1}}^{L}2\rho_{l_{1}l_{2}}\sqrt{p_{l_{1}}q_{l_{1}}p_{l_{2}}q_{l_{2}}}\beta_{l_{1}}\beta_{l_{2}}$. However, for a complex trait with polygenic genetic architecture, if the QTLs are randomly allocated along the genome, $\sigma_{A}^{2}=\Sigma_{l=1}^{L}2p_{l}q_{l}\beta_{l}^{2}$ (Supplementary Note V), a phenomenon that the between-locus covariances tradeoff. This is often true for a trait without ascertainment or selection. When each QTL is tagged perfectly and randomly allocated along the genome, Λ = 1. Equation (11) zeros out the Δ term and directly gives the unbiased estimate of twice negative of the additive variance. Removing the scale makes the heritability estimate unbiased. In practice, due to imperfect LD, the heritability is reduced to *h*^2^Λ.

In fact, the HE regression and the mixed model are equivalent and can agree on the heritability estimate (see Simulation III). However, this equivalence can be disturbed when QTL effects are not randomly distributed (Simulation IV).

### Extension to case-control GWAS data

Like the debut application of the original HE regression for schizophrenia (Elston et al., 1973), the IBS HE regression is also extended to case-control GWAS data in this study. However, due to scale issues and ascertainment (Dempster and Lerner, 1950; Falconer, 1966), the estimated heritability needs to be transformed to the liability scale, which is genetically meaningful for ascertained samples. One transformation was proposed by Lee et al. (2011), denoted here as Hong23. It is expressed as $h_{l}^{2}=h_{o}^{2}\frac{K(1-K)}{z^{2}}\frac{K(1-K)}{P(1-P)}$, in which *h*^2^_*l*_ is the heritability on the liability scale, *h*^2^_*o*_ is the heritability on the observed scale directly estimated based on the case-control data, *K* is the prevalence of the disease, *P* is the proportion of the cases in the data, and *z* is the height of the standard normal distribution in which the prevalence *K* is located.

Once the heritability is estimated by the HE regression on the observed scale with Hong23, it can be easily transformed from the observed scale to the liability scale. Simulation studies will be conducted to investigate whether the HE regression better estimates heritability than does the mixed linear model (Simulation IV).

In addition, *Y* in the HE regression can also be expressed as a cross-product, and then *E(b)* = −2*p_k_q_k_*τ^2^_*kl*_β^2^_*l*_, which is half that of Equation (7) (See Supplementary Note VI).

## Monte Carlo simulation results

In the Monte Carlo simulation, we will investigate the precision of the derived equations.

### Simulation I: one marker and one QTL [evaluation of Equation (7)]

This simulation investigated the accuracy of Equation (7) for a single-marker application. One thousand unrelated individuals were simulated. One marker and one QTL were simulated, both of which were equifrequent and biallelic. The heritability of the QTL was 0.5. The LD between the marker and the QTL was set at three levels: ρ = 0.25, ρ = 0.5, and ρ = 0.75. The single marker was used to construct the genetic relatedness, Ω. Then a single-marker-based HE regression was conducted. After standardizing the phenotype, the negative half of the regression coefficient returned the unbiased heritability estimate.

As indicated by Equation (7), given ρ = 0.25, ρ = 0.5, and ρ = 0.75, the regression coefficient expectation was −0.062, 0.125, and 0.57, respectively. After 100 rounds of simulation, the derived expectation of the regression coefficient, as well as the sampling variance (Table 6), were in good agreement with the simulation results listed in Table 7. This simulation indicates that the single-marker HE regression is a competitive tool for QTL mapping.

**Table 7:** **Simulation evaluations of Equation (7)**.

**LD**	**Analytical results^a^**	**Simulation results^b^**
ρ = 0.25	−0.062 (0.004)	−0.062 (0.0039)
ρ = 0.5	−0.25 (0.004)	−0.25 (0.0039)
ρ = 0.75	−0.56 (0.004)	−0.56 (0.0039)

a *The standard error was calculated*: 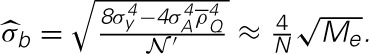. *Here N = 1000 and M_e_ = 1*.

b *The standard errors in parentheses indicate the mean of the standard error from 100 simulation replications*.

### Simulation II: statistical power of the single-marker He regression

For the single-marker HE regression, as the expectation and the sampling variance of the regression coefficient were already derived, a *t*-test could be constructed as $t=\frac{h^{2}\rho_{kl}^{2}}{2/N}$, in which the linkage disequilibrium between the *k*^*th*^ marker and the *l*^*th*^ QTL is ρ_*kl*_. When the sample size is sufficiently large, the *t*-test approaches the *z*-score distribution, and the non-centrality parameter of χ^2^_1_ is consequently $N\frac{h^{2}\rho_{kl}^{2}}{4}\cdot Nh^{2}\rho_{kl}^{2}\sim \chi_{1}^{2}$, a χ^2^-test with one degree of freedom. In Table 8, the required sample size to detect association with a SNP for a GWAS (type-I error rate of 10^−8^) and the required sample size to detect a QTL are indicated.

**Table 8:** **The sample size required for the single-marker HE regression to detect a QTL associated with the target marker**.

***h*^2^**	**ρ_*kl*_**		
	**0.25**	**0.5**	**0.75**
0.005	33,276	8,319	3,697
0.01	16,638	4,159	1,849
0.025	6,655	1,664	739
0.05	3,327	832	370

*Here the p-value cutoff was 10^−8^*.

In contrast, for a conventional one-marker association linear regression, *y*_i_ = μ + *b_k_s_ik_* + *e_i_*, if the phenotype and the genotypes are both standardized, *E*(*b_k_*) = ρ_*kl*_β_*l*_, and its standard error is $\sigma_{b_{k}}=\sqrt{\frac{\sigma_{e}^{2}}{N\sigma_{s_{k}}^{2}}}$, a *t*-test can be constructed as $t=\rho_{kl}\beta_{l}/\sqrt{\frac{\sigma^{2}\left(e\right)}{N\sigma^{2}\left(s_{k}\right)}}=\sqrt{\frac{Nh^{2}\rho_{kl}^{2}}{1-h^{2}\rho_{kl}^{2}}}$. Taking the square of the *t* statistic, the non-centrality parameter of $\frac{Nh^{2}\rho_{kl}^{2}}{1-h^{2}\rho_{kl}^{2}}\approx Nh^{2}\rho_{kl}^{2}\sim \chi_{1}^{2}$.

These two χ^2^ tests differ by the factor $N\frac{h^{2}\rho_{kl}^{2}}{4}$. Once $N>\frac{4}{h^{2}\rho_{kl}^{2}}$, the single-marker HE regression is more powerful than the conventional liner regression; otherwise, the conventional linear regression method is more powerful. As listed in Table 9, given that the heritability of a QTL is 0.01, if the LD between the target marker is low (ρ_*kl*_ = 0.25), medium (ρ_*kl*_ = 0.5), or high (ρ_*kl*_ = 0.75), the sample size required to allow HE to outperform the linear regression is 6400, 1600, and 712, respectively. If the heritability is even smaller, say *h*^2^ = 0.001, the required sample size is 12,800, 3200, and 1423 to make the HE regression more powerful under the low, medium, and high LD, respectively.

**Table 9:** **The required sample size that makes the HE regression more powerful than the conventional single-marker linear regression**.

***h*^2^**	**ρ_*kl*_**		
	**0.25**	**0.5**	**0.75**
0.005	12,800	3,200	1,423
0.01	6,400	1,600	712
0.025	2,560	640	285
0.05	1,280	320	143

Depending on the sample size, heritability, and LD patterns between the QTL and the target marker, the power of the HE regression may or may not be greater than the conventional linear regression. However, when the sample size is large, or the heritability of the QTL is large, HE regression is a more powerful tool for association studies. These results are based on the assumption that the real sampling variance agrees with the derived theoretical result.

### Simulation III: the all-marker He regression and the mixed linear model are equivalent [Δ = 0 in Equation (11)]

In this simulation, 100 equifrequent and biallelic QTLs were simulated, and the additive effect of each QTL was sampled from *N*(0, 1). Four LD levels (ρ*l*_1_, *l*_2_ = 0, 0.25, 0.5, 0.75) were adopted for each of two consecutive QTLs, and the effective number of markers decreased correspondingly (*M_e_* ≈ 100, 90, 61, 29). One thousand unrelated individuals were simulated, and the genetic relatedness of each pair of individuals was estimated on these 100 QTLs. The heritability of the simulated polygenic model was 0.5, which is calculated as $h^{2}=\frac{\sigma_{A}^{2}}{\sigma_{A}^{2}+\sigma_{e}^{2}}$. And $\sigma_{A}^{2}=\Sigma_{l=1}^{L}2p_{l}q_{l}\beta_{l}^{2}+\Sigma_{l_{1}=1}^{L}\Sigma_{l_{2}\neq l_{1}}^{L}2\rho_{l_{1}l_{2}}\sqrt{p_{l_{1}}q_{l_{1}}p_{l_{2}}q_{l_{2}}}\beta_{l_{1}}\beta_{l_{2}}$.

Both the HE regression and the mixed linear model were employed to estimate the additive variance component. The mixed linear model (Yang et al., 2010) can be expressed as *y*_*i*_ = μ + *x_ij_a_j_* + *e_i_*, where *y*_*i*_ is the phenotype of the *i*^*th*^ individual, μ is the mean, *x*_*ij*_ is the indicator variable with values of 0, 1, or 2 depending on the reference allele counts, and *e*_*j*_ is the residual. Restricted maximum likelihood (REML) was employed to estimate the variance components of the mixed linear model (Yang et al., 2010).

As shown in Table 10, the estimated heritability from either the HE regression or the mixed linear model was equal and not biased, demonstrating the equivalence between the HE regression and the mixed linear model when the QTLs are randomly distributed regardless of their pairwise LD.

**Table 10:** **Simulation evaluations of Equation (11) and comparison between the HE regression and the mixed linear model method (Δ = 0)**.

**LD (ρ)**	**Equation (11)^a^**	**HE results^b^**	**Mixed model results^c^**
ρ = 0	0.5 (0.020)	0.499 (0.020)	0.499 (0.041)
ρ = 0.25	0.5 (0.019)	0.500 (0.019)	0.501 (0.042)
ρ = 0.5	0.5 (0.016)	0.502 (0.015)	0.491 (0.043)
ρ = 0.75	0.5 (0.011)	0.488 (0.011)	0.508 (0.048)

a *Calculated given △ = 0*.

b,c *The standard errors in parentheses indicate the mean of the standard error from 100 simulation replications*.

*E*(*b*) = −2σ^2^_*A*_ Λ (ignoring Δ) sheds light on the inference of the general LD pattern between the tagged markers and the causal variance. $\Lambda =\frac{{\bar{\rho }}_{Q}^{2}}{{\bar{\rho }}_{M}^{2}}$, and ρ^2^_*M*_ can be estimated from markers. If the heritability of the trait is known (not likely though), it is possible to estimate ρ^2^_*Q*_. For example, the heritability of height is estimated at around 0.8 (Visscher et al., 2006; Perola et al., 2007) in linkage, but is 0.4 as estimated in an association study (Yang et al., 2010). If the estimate from linkage was considered to be the true heritability, $\hat{\Lambda }$ = 0.5. Assuming the effective number of markers is *M*_*e*_ = 10,000, ρ^2^_*M*_ = 0.0001, ρ^2^_*Q*_ = $\hat{\Lambda }$ρ^2^_*M*_ = 0.00005. The average absolute value of the LD between a QTL and a marker is 0.007.

### Simulation IV: when the He regression and the mixed linear model are not equivalent [when Δ ≠ 0 in Equation (11)]

The general setting for this simulation was similar to the last one, but the QTL effects were sorted such that the additive effects were increased along the simulated chromosomal segment. The covariance between any two QTLs can be predicted by *cov* 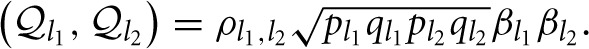. The heritability is defined as $h^{2}=\frac{\sigma_{A}^{2}}{\sigma_{A}^{2}+\sigma_{e}^{2}}$, in which $\sigma_{A}^{2}=\Sigma_{l=1}^{L}2p_{l}q_{l}\beta_{l}^{2}+\Sigma_{l_{1}=1}^{L}\Sigma_{l_{2}\neq l_{1}}^{L}2\rho_{l_{1}l_{2}}\sqrt{p_{l_{1}}q_{l_{1}}p_{l_{2}}q_{l_{2}}}\beta_{l_{1}}\beta_{l_{2}}$. Different from the last simulation, $\Delta =-2\Sigma_{k=1}^{M}(\Sigma_{l_{1}=1}^{L}\Sigma_{l_{2}\neq l_{1}}^{L}2\rho_{kl_{1}}\rho_{kl_{2}}\sigma_{l_{1}}\sigma_{l_{2}})/M\neq 0$.

With this set-up, which is not likely to be true in practice but illustrates an extreme case, the HE regression and the mixed model gave very different estimations. With increased correlation between markers, the HE gave inflated estimates and the mixed model gave deflated estimates. Although both methods gave biased estimates, Equation (11) still could predict the results of the HE regression correctly (See Table 11).

**Table 11:** **Simulation evaluations of Equation (11) when the covariance summation is not zero (Δ ≠ 0)**.

**LD (ρ)**	**Equation (11)^a^**	**HE results^b^**	**Mixed model results^c^**
ρ = 0	0.500 (0.020)	0.497 (0.020)	0.499 (0.041)
ρ = 0.25	0.715 (0.019)	0.712 (0.019)	0.414 (0.041)
ρ = 0.5	0.853 (0.015)	0.850 (0.015)	0.347 (0.043)
ρ = 0.75	0.878 (0.011)	0.881 (0.011)	0.291 (0.048)

a *Calculated given △ ≠ 0*.

b,c *The standard errors in parentheses indicate the mean of the standard error from 100 simulation replications*.

### Simulation V: application to case-control data

The HE regression was applied to case-control data. A polygenic model of *L* equifrequent diallelic QTLs was simulated, and each locus was in Hardy–Weinberg equilibrium and any pair of QTLs was in linkage equilibrium. The heritability on the liability scale was *h*^2^_*l*_, the heritability on the liability scale. The effect of each QTL was sampled from *N*(0, σ^2^_*b*_), and σ^2^_*b*_ = *h_l_*^2^/[2 × *p* × (1 − *p*) × *L*], in which *p* = 0.5. The phenotype of each individual under the liability scale was scaled to unit. The ascertainment of cases on the liability scale was *K*. Individuals were sampled from the described reference population until 1000 cases and 1000 controls were recruited.

The heritability on the liability scale was 0.5. In order to cover a broad range of scenarios, three levels of QTL number, *L* = 100, 1000, and 10,000, and three levels of disease prevalence at the population level, *K* = 0.1, 0.01, and 0.001, were adopted. Nine scenarios were simulated in total, and 30 independent simulation replications were implemented for each scenario.

The genetic relationship matrix was constructed using all individuals and the allele frequencies were estimated from the sample. The genetic additive variance components were estimated with the HE regression and the mixed model method. As the directly estimated variance component was on the observed scale and could be greater than 1, we employed both the REML and non-constrained REML for mixed model methods, which allowed the heritability to be greater than 1.

**Figure 1:**
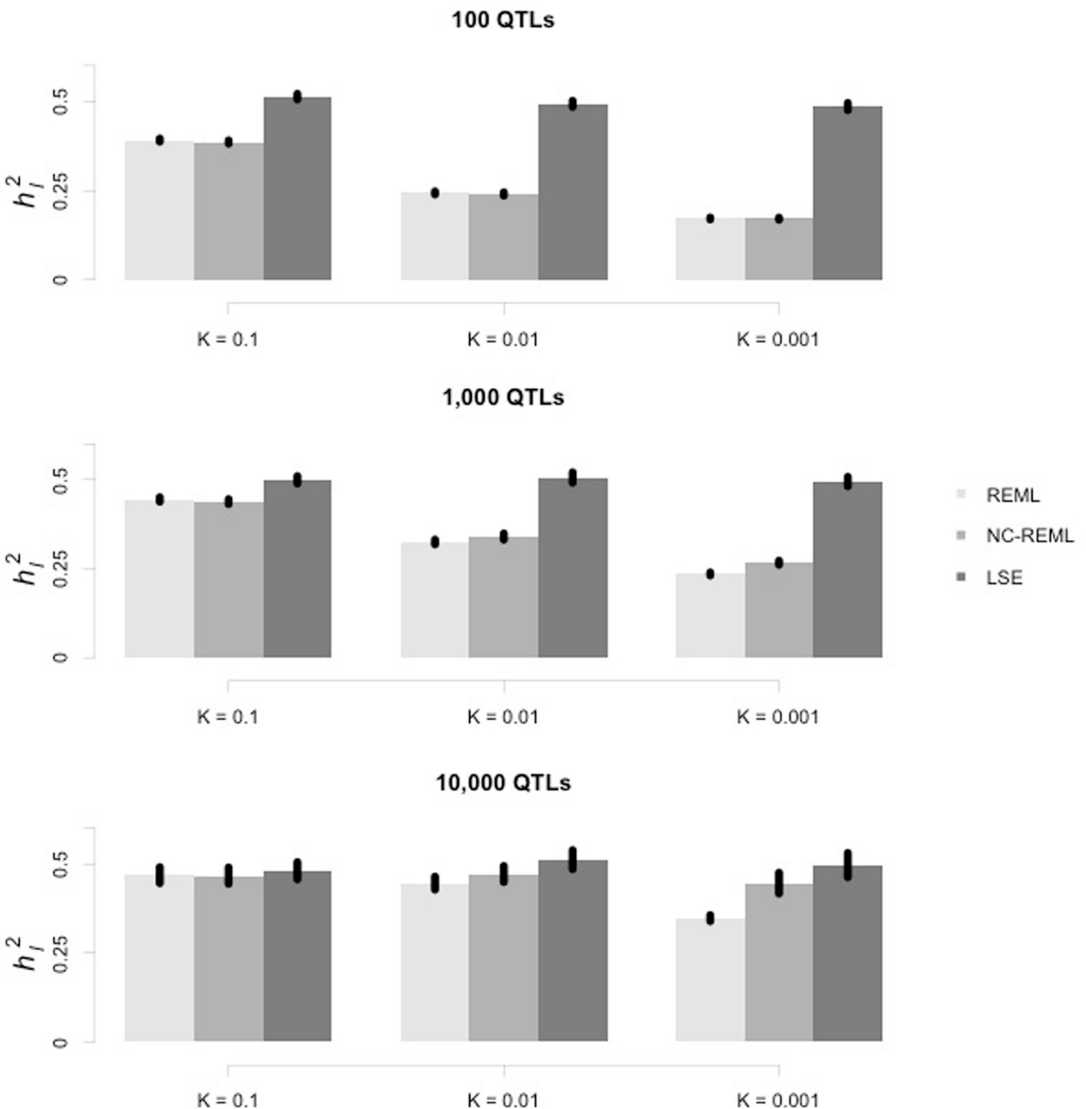
**Estimation of heritability on the liability scale using the HE regression and mixed linear model methods**. In each row, from left to right, each panel represents the case-control sample simulated under the same heritability on the liability scale (*h*^2^_*l*_) but with different prevalence. In each panel, the vertical axis indicates the estimated heritability on the liability scale (*h*^2^_*l*_), whereas the horizontal axis indicates which of the three methods (REML, non-constrained REML, and HE regression [least square estimate]) was used. The standard error of the mean (SEM) is indicated at the top of each bar.

As illustrated in Figure 1, the estimated *h*^2^_*l*_ was compared across all three methods. In general the HE regression resulted in a more precise estimate than that of the REML and non-constrained REML. For the mixed model methods, either with or without constraints, REML often underestimated the variance components. The bias was caused by two factors: the number of QTLs (in each row panel) and the prevalence of the disease (in each column panel). With fewer QTLs, a lower prevalence could exacerbate underestimation by the mixed model.

### Conclusion

The analytical results summarized in Table 6 were evaluated using Monte Carlo simulation, and were highly precise in general. The single-marker HE regression is a competitive tool for QTL mapping, particularly with a large sample size (Simulations I, II). The HE regression and the mixed model method were equivalent, with both providing a precise heritability estimate for a typical polygenic trait (Simulation III). However, if QTL effects are correlated, neither the HE regression nor the mixed model method gave an unbiased estimate (Simulation IV). For case-control studies, the HE regression should be preferred in general (Simulation V).

## Genetic analysis repository (GEAR)

In order to facilitate application of the HE regression method to estimate complex trait heritability, GEAR software was developed. GEAR was developed on Java and can run across many operating systems, such as Windows, Mac, and Linus/Unix, as long as a Java virtual machine is available. GEAR has been demonstrated to function in the following situations.

1. It can generate genetic relatedness of unrelated individuals, as formulated in Equation (3), based on whole-genome markers.
2. It can estimate the effective number of markers based on a genetic-relatedness matrix.
3. It can estimate heritability with the HE regression. GEAR can read genotype data saved in PLINK binary format (Purcell et al., 2007).

GEAR can be downloaded from the website: https://sourceforge.net/projects/gbchen/files/GEAR/

The online GEAR manual can be found at https://sourceforge.net/p/gbchen/wiki/GEAR/

## Discussion

Historically, linkage was the major tool for QTL mapping of complex traits since the 1970s, which was gradually replaced by association analysis when GWAS became popular (The Wellcome Trust Consortium, 2007). The transmission/disequilibrium test (TDT; Ott, 1989; Spielman et al., 1993) triggered the transition from linkage to association for family-based studies. In the year 2000, generalized TDT was proposed (Laird et al., 2000), which is robust for population stratification. Shortly after that, population-based design emerged as the major flow in genetic data, and GWAS became the leading method for estimating heritability up until now. Extension of the original HE regression to association studies can be seen as an effort to increase the diversity of GWAS analysis tools.

In this study, we established a theory for a modified HE regression, in which IBS scores replace IBD scores. Although IBS is used to detect IBD in linkage studies (Lange, 1986a,b; Bishop and Williamson, 1990), it is considered to be a way of inferring IBD for relatives, such as sib pairs, when founder genotypes are unavailable. In this study, IBS served as the key concept to detect association of unrelated samples rather than relatives. Linkage and association have both been proposed to estimate heritability of complex traits. For example, for height, the heritability estimated from linkage studies is around 0.8 (Visscher et al., 2006; Perola et al., 2007), but around 0.4 from association studies (Yang et al., 2010). Thus, far there is no clear conclusion regarding the fundamental difference between the heritability estimated from these two kinds of methods. Despite their mathematical similarity, application, and interpretation differences should be appreciated.

Under various scenarios, the mathematical expectations of the regression coefficients, as well as the sampling variances, were derived. There is substantial mathematical similarity between the IBD HE regression and the IBS HE regression. For example, for both models under the single-marker scenario, their regression coefficients can be expressed in a unified form, *E*(*b*) = − 2ρ^2^ σ^2^_*A*_. As these two models are based on different genetic mechanisms, the interpretations of their respective regression coefficients are reasonably different. In the IBD-based HE regression, *E*(*b*) = −2(1 − 2*c*)^2^ σ*_A_l__*^2^, 1 − 2*c* ranges from 0 to 1; whereas in the IBS-based HE regression, *E*(*b*) = −2τ^2^_*kl*_ σ*_A_l__*^2^, in which τ*_kl_*, ranges from −1 to 1. As the values of *r* and *R* rely upon the allele frequencies of the biallelic marker and the biallelic QTL, they reach either −1 or 1 only given that the marker has the same allele frequency as that of the QTL. However, after taking the square, both (1 − 2*c*)^2^ and τ^2^ lie between 0 and 1, inclusive.

Equation (11), *E*(*b*) = −2σ^2^_*A*_ Λ (ignoring Δ), provides a possible way to estimate the LD pattern between causal loci and markers. If the true heritability is not readily known, it is possible to estimate ρ^2^_*Q*_, the average LD between QTLs and markers. As demonstrated in simulation III, it may be possible to estimate ρ^2^_
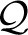
_. However, the causal loci can be in any possible form, such as SNPs, chromatin markers, or methylation markers, and different methods capture genetic variation in different forms. In practice, the obstacle in estimating ρ^2^_
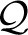
_ lies in how heritability estimated from different methods, such as linkage and association, or genotyping platforms, such as SNP markers and methylation markers, can be connected to each other. Equation (11) sheds light on the investigation for how QTLs are distributed along the genome.

Application of the HE regression to heritability estimation of complex traits revealed that the HE regression seems to be equivalent to the mixed model approaches in general (Δ = 0). A similar equivalence was previously established for linkage analysis (Sham and Purcell, 2001). However, for GWAS data, it should be noted that the equivalence is conditional on the genetic architecture of a trait. As indicated in the simulation, the equivalence stands only for typical polygenic genetic architecture, which may be true for many traits without ascertainment or selection, such as height (Yang et al., 2010). However, when substantial covariance exists between causal loci, the equivalence does not stand and neither the HE regression nor the mixed model method gave unbiased estimates. The equivalence may break down under other circumstances that have not been investigated. In real studies, this kind of covariance may be a result of selection in active regions, such as HLA loci, which harbors many signals; then, the HE regression and the mixed model estimates may differ. The equivalence may break down under other circumstances that have not been investigated in this study.

In GWAS, many samples are collected for complex diseases, which are often in a case-control design. Complex disease prevalence is often low; consequently, the cases are under strong ascertainment, which disrupts the assumptions underlying the mixed linear model. As observed in the simulation studies, the HE regression is more precise in estimating heritability than the mixed linear model for case-control studies across a broad range of scenarios. Use of HE regression is advantageous when the disease prevalence is low and the number of causal loci is few. In their original work, Lee et al. (2011) assumed an infinitesimal model of complex diseases. However, when this assumption was disrupted during simulation (likely in practice as well), the mixed linear model method gave biased estimates of heritability. Thus, whenever possible, the HE regression method is preferable to estimate heritability of complex traits.

As derived in this work, the HE regression and the mixed model method are equivalent under polygenic genetic architecture. In other words, when the estimates generated by these two methods significantly differ for the same data, caveats should be presented. As investigated in the simulation, the real heritability may lie between the estimates of these two methods. Speed et al. (2012) previously investigated the assumptions underlying the mixed model method and proposed alternative weighting methods to adjust the heritability estimation. However, as their weighting method depends on genetic architecture, which is often unknown, it is difficult to justify which weighting method is appropriate to adopt for certain data (Gusev et al., 2013). Thus, simply comparing the estimates from the HE regression and the mixed model method may offer an alternative way of justification.

It should be noted that the HE regression method is on the basis of the least square framework rather than the maximal likelihood framework as many mixed model based on (Yang et al., 2010; Speed et al., 2012; Lee et al., 2013). As a numerical method, maximal likelihood methods give estimates optimizing the likelihood under the assumptions, which may break down in practice. Given recent interests in comparing estimates with or without imputation for the genome (Gusev et al., 2013), controversial results have been observed. It is not sure what the increased or decreased estimation of heritability indicates after imputation. A reasonable guess will be that the local covariance structure, as indicated in Equation (11), changes and eventually bring out different estimates. The proposed IBS HE regression, which depends on fewer assumptions compared with maximal likelihood methods, may help melt the controversy.

In practice, undocumented relatedness may creep into samples, and eventually bring about suspiciously high relatedness. As discussed previously (Powell et al., 2010), Equation (3) gives a score of 0 for a pair of unrelated individuals, 0.5 for first-degree relatives, and 1 for duplicated individuals or monozygotic twins. It seems easy to eliminate related individuals if a cutoff, say a relatedness of less than 0.05, is applied to the sample. For association studies, population stratification may increase false positive rates. To reduce the threat of population stratification, phenotypes can be adjusted by principal components (Price et al., 2006) and then fit into the HE regression. If a sample is admixed, the power of the HE regression may be reduced if, in the ancestral populations, the allele frequency spectrums are different from each other or genetic heterogeneity exists in the genetic architecture of the underlying trait in question. More investigation will be required to overcome this challenge.

The variance components have often been estimated via REML (Yang et al., 2010; Lee et al., 2011). Given its various merits, REML is computationally expensive, particularly for large sample sizes. The computational complex is on the scale of *O*(*tN*^3^), which indicates that it is cubic to the sample size and *t* rounds of iterations. The time complex of the HE regression is far lower, asymptotically *O*(2*N*^2^), given two parameters, the intercept and the regression coefficient, included in the model. Given the large sample sizes often employed in GWAS, the computational burden can be dramatically reduced. Although the HE regression method is derived on a simple-regression scenario, its extension to a multiple-regression scenario is straightforward. For instance, the genetic relatedness between each pair of individuals can be constructed on each chromosome and then all chromosome-based relatedness scores can be fit into the regression framework. In addition, the difference between a pair of phenotypes can also be expressed as a cross product and squared sum (Sham and Purcell, 2001).

## Author contributions

Guo-Bo Chen conceived the study, derived the equations, conducted the simulation studies, and calculated the analytical results. Guo-Bo Chen developed and maintained the GEnetic Analysis Repository (GEAR) software. Guo-Bo Chen wrote the manuscript.

### Conflict of interest statement

The author declares that the research was conducted in the absence of any commercial or financial relationships that could be construed as a potential conflict of interest.
