# Supplementary material for: Estimating heritability of complex traits from genome-wide association studies using IBS-based Haseman–Elston regression
Source: Front Genet. 2014 Apr 30;5:107. doi: 10.3389/fgene.2014.00107 (PMC4012219; doi:10.3389/fgene.2014.00107)
Supplement: Supplementary file 1 [file DataSheet1.PDF]

Supplementary materials

Table S1 Conditional probabilities of four haplotype phases consisting of a biallelic loci pair

		The lth locus		
		a_l	A_l	Marginal probability
The kth locus	a_k	$r_{kl} = q_l + \frac{D_{kl}}{q_k}$	$1 - r_{kl} = p_l - \frac{D_{kl}}{q_k}$	q_k
	A_k	$1 - R_{kl} = q_l - \frac{D_{kl}}{p_k}$	$R_{kl} = p_l + \frac{D_{kl}}{p_k}$	p_k
Marginal probability		q_l	p_l	

Notes:

$r_{kl} = p(a_l|a_k)$ and $R_{kl} = p(A_l|A_k)$ are the conditional probabilities of the two coupling haplotypes.

D_{kl} measures linkage disequilibrium for the two loci, $D_{kl} = f_{a_k a_l} - q_k q_l$, in which $f_{a_k a_l}$ is the frequency of the haplotype consisting of a_k and a_l. If $D_{kl} > 0$, $D_{kl} \leq \min(q_k p_l, p_k q_l)$; if $D_{kl} < 0$, $D_{kl} \leq \min(q_k q_l, p_k p_l)$.

Table S2 The joint distribution of $E(\Omega_{ij})$ and $E(Y_{ij} \mid x_i, x_j)$ for one marker and multiple QTLs

					Individual i		
				Genotype (x_i)	$a_k a_k$	$A_k a_k$	$A_k A_k$
				s_{ik}	$\frac{-2p_k}{\sqrt{2p_k q_k}}$	$\frac{q_k - p_k}{\sqrt{2p_k q_k}}$	$\frac{2q_k}{\sqrt{2p_k q_k}}$
				$E(y_i \mid x_{ik})$	$\Sigma_l^L(1-2r_{kl})\beta_l$	$\Sigma_l^L(R_{kl}-r_{kl})\beta_l$	$\Sigma_l^L(2R_{kl}-1)\beta_l$
	Genotype (x_j)	s_{jk}	$E(y_j \mid x_{jk})$	Frequency	q_k^2	$2p_k q_k$	p_k^2
Individual j	$a_k a_k$	$\frac{-2p_k}{\sqrt{2p_k q_k}}$	$\Sigma_l^L(1-2r_{kl})\beta_l$	q_k^2	$\frac{4p_k^2}{2p_k q_k}$	$\frac{-2p_k(q_k - p_k)}{2p_k q_k}$	$\frac{-4p_k q_k}{2p_k q_k}$
					0	$(\Sigma_l^L -\tau_{kl}\beta_l)^2$	$4(\Sigma_l^L -\tau_{kl}\beta_l)^2$
					q_k^4	$2p_k q_k^3$	$p_k^2 q_k^2$
	$A_k a_k$	$\frac{q_k - p_k}{\sqrt{2p_k q_k}}$	$\Sigma_l^L(R_{kl}-r_{kl})\beta_l$	$2p_k q_k$	$\frac{-2p_k(q_k - p_k)}{2p_k q_k}$	$\frac{(q_k - p_k)^2}{2p_k q_k}$	$\frac{2q_k(q_k - p_k)}{2p_k q_k}$
					$(\Sigma_l^L \tau_{kl}\beta_l)^2$	0	$(\Sigma_l^L -\tau_{kl}\beta_l)^2$
					$2p_k q_k^3$	$4p_k^2 q_k^2$	$2p_k^3 q_k$
	$A_k A_k$	$\frac{2q_k}{\sqrt{2p_k q_k}}$	$\Sigma_l^L(2R_{kl}-1)\beta_l$	p_k^2	$\frac{-4p_k q_k}{2p_k q_k}$	$\frac{2q_k(q_k - p_k)}{2p_k q_k}$	$\frac{4q_k^2}{2p_k q_k}$
					$4(\Sigma_l^L \tau_{kl}\beta_l)^2$	$(\Sigma_l^L \tau_{kl}\beta_l)^2$	0
					$p_k^2 q_k^2$	$2p_k^3 q_k$	p_k^4

Notes:

$x_{.k}$ represents the standardized genotypes of the kth locus.

The nine highlighted cells, each of which has three terms, list the expected value of Ω_{ij} and Y_{ij}. In each cell, three terms from the top to the bottom are $\Omega_{ij} = s_{ik}s_{jk}$, $E(Y_{ij} \mid x_{ik}, x_{jk}) = [E(y_i \mid x_{ik}) - E(y_j \mid x_{jk})]^2$, and their respective probabilities.

$\tau_{kl} = 1 - r_{kl} - R_{kl}$. L is the number of QTLs.

Supplementary Note I: The covariance between two relatedness fractions (represented in Pearson's correlation)

Let Ω_{ijk} and Ω_{ijl} be the relatedness fractions for individuals i and j over the kth and the lth markers. The covariance between Ω_{ijk} and Ω_{ijl} can be written as $cov(\Omega_{ijk}, \Omega_{ijl}) = E(\Omega_{ijk}\Omega_{ijl})$ because the mean of both Ω_{ijk} and Ω_{ijl} is zero. As individuals are unrelated, $E(\Omega_{ijk}\Omega_{ijl}) = E(s_{ik}s_{jk}s_{il}s_{jl}) = E(s_{ik}s_{il})E(s_{jk}s_{jl})$, in which s represents the standardized genotype.

$E(s_{ik}s_{il}) = cov(\Omega_{ik}, \Omega_{il}) = \rho_{kl}$, in which ρ_{kl} is defined as the correlation between the kth and the lth markers.

Thus, $cov(\Omega_{ijk}, \Omega_{ijl}) = \rho_{kl}^2$.

Supplementary Note II: the effective number of markers, M_e

Two methods are used to evaluate the effective number of markers on a GWAS chip: the first is a chi-square test method and the second is a genetic relatedness score matrix. In this note, we demonstrate that these two methods are mathematically equivalent.

Method 1: simulation Chi-square method

The simulation algorithm used for evaluation was introduced in the context of risk prediction (supplementary page 36, Purcell et al., 2009). The simulation algorithm is as below.

Take all N individuals, each of which has M genotype markers. Then perform the calculations as follows:

1 Randomly assign 0 or 1 to each individual with probability p, say 0.5.

2 Conduct GWAS and calculate a chi-square statistic for each marker.

3 Take the sum of all chi-square statistics, denoted as CS_i for the ith simulation.

Repeat 1-3 for K rounds of simulation and calculate the variance of CS_i, denoted as V_{cs}. The effective number of markers is consequently defined as

$$M_e = M \times [\frac{2M}{V_{cs}}]$$

M_e is interpreted as the number of independent markers in terms of the chi-square distribution.

Method 2: the genetic relationship matrix method

1 The regression of y on the ith marker

$$y = a_i + b_i x_i + e$$

when $y \sim N(a + b_i x_i, \sigma_e^2)$ and $\sigma_e^2 = 1$, and x_i is the standardized genotype.

$\hat{b_i} = \frac{cov(x_i,y)}{var(x_i)}$, $var(b_i) = \sigma_e^2/var(x_i)$, and the t-test statistic is $t_i = \frac{\hat{b_i}}{\hat{\sigma}_{b_i}} = \frac{cov(x_i,y)}{var(x_i)} / \sqrt{\frac{\sigma_e^2}{var(x_i)}}$.

Given the large sample size, which provides sufficient degrees of freedom, the t-test nearly follows the standard normal distribution. $t_i^2 = \frac{cov^2(x_i,y)}{var(x_i)\sigma_e^2}$ follows a chi-square distribution with 1 degree of freedom. Furthermore, because $E(\sigma_e^2) = 1$ and $var(y) = 1$, we can rewrite $t_i^2 = \frac{cov^2(x_i,y)}{var(x_i)\sigma_e^2} = \frac{cov^2(x_i,y)}{var(x_i)var(y)} = \rho_i^2 \sim \chi_1^2$ when ρ_i is the correlation between the ith marker and the phenotype.

2 The regression of y on the jth marker

$$y = a_j + b_j x_j + e$$

The correlation between x_i and x_j is λ_{ij}, in which, it should be noted, λ_{ij} is the mathematical expectation of the ith and j^{th} markers. We can write $x_j = \lambda_{ij} x_i + \sqrt{1 - \lambda_{ij}^2}\varepsilon$, when ε follows the standard normal distribution, and $var(x_j) = 1$, $cov(x_i, x_j) = \lambda_{ij}$,

$\hat{b_j} = \frac{cov(\lambda_{ij}x_i + \sqrt{1-\lambda_{ij}^2}\varepsilon, y)}{var(x_i)} = \lambda_{ij} b_i + \sqrt{1 - \lambda_{ij}^2} b_z$. ε is not correlated with y either because y is randomly simulated, $\hat{b_j} = \lambda_{ij} b_i$

$t_j^2 = \lambda_{ij}^2 \rho_i^2 \sim \chi_1^2$.

Now, $var(t_i^2 + t_j^2) = var(t_i^2) + 2cov(t_i^2, t_j^2) + var(t_j^2) = 2 + 2cov(t_i^2, t_j^2) + 2$

What $cov(t_i^2, t_j^2) = \lambda_{ij}^2 cov(t_i^2, t_i^2) = \lambda_{ij}^2 \times 1 \times \sqrt{var(t_i^2)var(t_j^2)} = 2\lambda_{ij}^2$.

$var\left(t_i^2 + t_j^2\right) = 2(\lambda_{ii}^2 + 2\lambda_{ij}^2 + \lambda_{jj}^2)$. If we expand it further, it becomes $var(\Sigma_i t_i^2) = 2\left(\Sigma_i \lambda_{ii}^2 + \Sigma_i \Sigma_{j\neq i} \lambda_{ij}^2\right) = 2(M + \Sigma_i^M \Sigma_{j\neq i}^M \lambda_{ij}^2)$ **(Equation A1)**

$M_e = M \times \left[\frac{2M}{V_{cs}}\right] = \frac{M^2}{M + \sum_{i=1}^{M} \sum_{j\neq i}^{M} \lambda_{ij}^2}$ **(Equation A2)**.

When every pair of markers is in linkage equilibrium with each other, $M_e = M$.

Supplementary Note III: The derivation of $\boldsymbol{var\left[(y_i - y_j)^2\right]}$.

$$
\begin{aligned}
var(Y_{ij}) = var\left[(y_i - y_j)^2\right] &= var(y_i^2 + y_j^2 - 2y_iy_j) \\
&= var(y_i^2) + var(y_j^2) + 4var(y_iy_j) + 2cov(y_i^2, y_j^2) - 4cov(y_i^2, y_iy_j) \\
&\quad - 4cov(y_j^2, y_iy_j)
\end{aligned}
$$

$$var(y_i^2) = var(y_j^2) = E(y_i^4) - E^2(y_i^2) = 4\mu_y^2\sigma_y^2 + 2\sigma_y^4$$

$$
\begin{aligned}
var(y_iy_j) &= E(y_i^2y_j^2) - E^2(y_iy_j) = E(y_i^2)E(y_j^2) - E^2(y_i)E^2(y_j) = (\mu_y^2 + \sigma_y^2)^2 - \mu_y^2\mu_y^2 \\
&= 2\mu_y^2\sigma_y^2 + \sigma^4
\end{aligned}
$$

$$cov(y_i^2, y_j^2) = E(y_i^2y_j^2) - E(y_i^2)E(y_j^2) = E(y_i^2)E(y_j^2) - E(y_i^2)E(y_j^2) = 0$$

$$
\begin{aligned}
cov(y_i^2, y_iy_j) &= E(y_i^2y_iy_j) - E(y_i^2)E(y_iy_j) = E(y_i^3)E(y_j) - E(y_i^2)E(y_i)E(y_j) \\
&= E(y_j)[E(y_i^3) - E(y_i^2)E(y_i)] = \mu_y[\mu_y^3 + 3\mu_y\sigma_y^2 - (\mu_y^2 + \sigma_y^2)\mu_y] = 2\mu_y^2\sigma_y^2
\end{aligned}
$$

Eventually, $var(Y_{ij}) = 8\sigma_y^4$.

The moment of y, which follows a normal distribution with a mean of μ_y and deviation of σ_y^2 at different orders can be found in the table below.

Order	Moment of normal distribution
1	μ_y
2	$\mu_y^2 + \sigma_y^2$
3	$\mu_y^3 + 3\mu\sigma_y^2$
4	$\mu_y^4 + 6\mu_y^2\sigma_y^2 + 3\sigma_y^4$

Supplementary Note IV: $\Delta \approx 0$ for GWAS data

As derived in Scenario II, the regression coefficient is:

$E(b) = \{\frac{-2\Sigma_{k=1}^{M}\Sigma_{l_1=1}^{L}\Sigma_{l_2=1}^{L}\rho_{kl_1}\rho_{kl_2}\sigma_{l_1}\sigma_{l_2}}{M}\}/\{\frac{\Sigma_{k=1}^{M}\Sigma_{l=1}^{M}\rho_{kl}^2}{M^2}\}$ **(Equation A3)**.

Its numerator is

$-2\Sigma_{k=1}^{M}\left[\Sigma_{l=1}^{L}\rho_{kl}^2\sigma_l^2 + \Sigma_{l_1=1}^{L}\Sigma_{l_2\neq l_1}^{L}\rho_{kl_1}\rho_{kl_2}\sigma_{l_1}\sigma_{l_2}\right]/M$ **(Equation A4)**

in which the first summation term is the correlation between the kth marker and a QTL and the second term is the correlation between any pair of QTLs dependent on the kth marker. The elements in the brackets can also be written in a quadratic form

$$\mathbb{Q}^T A_k \mathbb{Q} = \Sigma_{l=1}^{L}\rho_{kl}^2\sigma_l^2 + \Sigma_{l_1=1}^{L}\Sigma_{l_1\neq l_2}^{L}\rho_{kl_1}\rho_{kl_2}\sigma_{l_1}\sigma_{l_2}$$

in which $\mathbb{Q} = [\sigma_1, \sigma_2, \dots, \sigma_L]$, and

$$A_k = \begin{bmatrix} \rho_{k1}^2 & \cdots & \rho_{k1}\rho_{kL} \\ \vdots & \ddots & \vdots \\ \rho_{kL}\rho_{k1} & \cdots & \rho_{kL}^2 \end{bmatrix}$$

is the variance-covariance matrix for the QTLs upon the kth marker, in which the diagonal elements are the LD between the kth marker and the lth QTL in terms of squared Pearson's correlation, and the off-diagonal elements are covariance of any pair of QTLs dependent on the kth marker.

If the QTLs are randomly distributed along the genome, we can split the first summation term in Equation A4, $\Sigma_{l=1}^{L}\rho_{kl}^2h_l^2 = \frac{[\Sigma_{l=1}^{L}\sigma_l^2]}{L}[\Sigma_{l=1}^{L}\rho_{kl}^2] = \frac{\sigma_A^2}{L}(\Sigma_{l=1}^{L}\rho_{kl}^2)$, in which $\sigma_A^2 = \Sigma_{l=1}^{L}\sigma_l^2$ represents the sum of the additive variance over the QTLs. Thus, $\Sigma_{l=1}^{L}\rho_{kl}^2\sigma_l^2 = \frac{\sigma_A^2}{L}(\Sigma_{l=1}^{L}\rho_{kl}^2)$. As the QTL effects are independent of the correlation between the QTL and the markers, we can split them $\Sigma_{l_1=1}^{L}\Sigma_{l_1\neq l_2}\rho_{kl_1}\rho_{kl_2}\sigma_{l_1}\sigma_{l_2} = (\Sigma_{l_1=1}^{L}\Sigma_{l_1\neq l_2}^{L}\sigma_{l_1}\sigma_{l_2})(\frac{\Sigma_{l_1=1}^{L}\Sigma_{l_1\neq l_2}^{L}\rho_{kl_1}\rho_{kl_2}}{L(L-1)})$. The first term $\Sigma_{i=1}^{L}\Sigma_{i\neq j}^{L}\rho_{ik}\rho_{jk} = 0$, if the covariance between a marker and a QTL is zero; the second term should also be zero if there are no forces bringing about correlation between LD and QTL effects. So, $\Sigma_{l_1=1}^{L}\Sigma_{l_1\neq l_2}\rho_{kl_1}\rho_{kl_2}\sigma_{l_1}\sigma_{l_2} = 0$. As the effects associated with each marker can be represented in such a matrix, the total numerator encapsulates such matrices into M. $L(L-1)M$ elements can be eliminated from the numerator in Equation 11.

Eventually the numerator of Equation 11 can be simplified as

$$\left\{\frac{-2\Sigma_{k=1}^{M}\left[\Sigma_{l=1}^{L}\rho_{kl}^2\sigma_l^2 + \Sigma_{l_1=1}^{L}\Sigma_{l_2\neq l_1}^{L}\rho_{kl_1}\rho_{kl_2}\sigma_{l_1}\sigma_{l_2}\right]}{M}\right\} = -2\frac{\sigma_{\mathrm{A}}^2}{L}\frac{\Sigma_{k=1}^{\mathrm{M}}\Sigma_{i=1}^{L}(\rho_{ik}^2)}{M}.$$

Then Equation A4 becomes $E(b) = -2\sigma_{\mathrm{A}}^2\Lambda$.

Supplementary Note V: Additive genetic variance structure of whole genome data

Let $y_i = \mu_y + \Sigma_l^L \beta_l x_{il} + e_i$, and $var(y_i) = var\left(\Sigma_l^L \beta_l x_{il}\right) + var(e_i)$. The additive genetic variance is $\sigma_A^2 = \Sigma_l^L 2p_l q_l \beta_l^2 + \Sigma_{l_1}^L \Sigma_{l_2 \neq l_1}^L 2\rho_{l_1 l_2} \sqrt{p_{l_1} q_{l_1} p_{l_2} q_{l_2}} \beta_{l_1} \beta_{l_2}$, in which $\rho_{l_1 l_2}$ is the correlation between a pair of QTLs. The first summation quantifies the within-locus additive genetic variance, whereas the second summation quantifies the between-locus additive genetic variance due to linkage-disequilibrium. Although the additive genetic variance is raised by both of the summations among limited loci, it should be noted that for a quantitative trait without selection, the second summation is neutralized to be zero. Thus, for a quantitative trait, $h^2 = \sigma_A^2 \approx \Sigma_l^L 2p_l q_l \beta_l^2$, the within-locus variance alone almost entirely determines the additive variance component. This is an important property of the heritability estimate on the whole-genome scale.

Supplementary Note VI: Regression coefficient expectation when the phenotype is a cross-product

					Individual i		
				Genotype	$a_k a_k$	$A_k a_k$	$A_k A_k$
				s_{ik}	$\frac{-2p_k}{\sqrt{2p_k q_k}}$	$\frac{q_k - p_k}{\sqrt{2p_k q_k}}$	$\frac{2q_k}{\sqrt{2p_k q_k}}$
	Genotype (x_j)	s_{jk}	$E(y_j \mid x_{jk})$	Frequency	$(1-2r_{kl})\beta_l$	$(R_{kl}-r_{kl})\beta_l$	$(2R_{kl}-1)\beta_l$
Individual j	$a_k a_k$	$\frac{-2p_k}{\sqrt{2p_k q_k}}$	$(1-2r_{kl})\beta_l$	q_k^2	$\frac{4p_k^2}{2p_k q_k}$	$\frac{-2p_k(q_k-p_k)}{2p_k q_k}$	$\frac{-4p_k q_k}{2p_k q_k}$
					$(1-2r_{kl})^2\beta_l^2$	$(1-2r_{kl})(R_{kl}-r_{kl})\beta_l^2$	$(2R_{kl}-1)(1-2r_{kl})\beta_l^2$
					q_k^4	$2p_k q_k^3$	$p_k^2 q_k^2$
	$A_k a_k$	$\frac{q-p}{\sqrt{2pq}}$	$(R_{kl}-r_{kl})\beta_l$	$2p_k q_k$	$\frac{-2p_k(q_k-p_k)}{2p_k q_k}$	$\frac{(q_k-p_k)^2}{2p_k q_k}$	$\frac{2q_k(q_k-p_k)}{2p_k q_k}$
					$(1-2r_{kl})(R_{kl}-r_{kl})\beta_l^2$	$(R_{kl}-r_{kl})^2\beta_l^2$	$(R_{kl}-1)(R_{kl}-r_{kl})\beta_l^2$
					$2p_k q_k^3$	$4p_k^2 q_k^2$	$2p_k^3 q_k$
	$A_k A_k$	$\frac{2q}{\sqrt{2pq}}$	$(2R_{kl}-1)\beta_l$	p_k^2	$\frac{-4p_k q_k}{2p_k q_k}$	$\frac{2q_k(q_k-p_k)}{2p_k q_k}$	$\frac{4q_k^2}{2p_k q_k}$
					$(2R_{kl}-1)(1-2r_{kl})\beta_l^2$	$(2R_{kl}-1)(R_{kl}-r_{kl})\beta_l^2$	$(2R_{kl}-1)^2\beta_l^2$
					$p_k^2 q_k^2$	$2p_k^3 q_k$	p_k^4

Notes:

$s_{.k}$ represents the standardized genotypes of the kth locus.

The nine highlighted cells, each of which has three elements, list the expected value of $E(X_{ij})$ and $E\left(Y_{ij} \middle| x_{ik}, x_{jk}\right)$. In each highlighted cell, three terms from the top to the bottom are $\Omega_{ij} = s_{ik}s_{jk}$, $E\left(Y_{ij} \middle| x_{ik}, x_{jk}\right) = \left[E(y_i|x_{ik}) - E\left(y_j \middle| x_{jk}\right)\right]^2$, and their respective frequencies.

$\tau_{kl} = 1 - r_{kl} - R_{kl}$.

$$E(b) = \left[\frac{-2p_k}{\sqrt{2p_k q_k}}(1-2r_{kl})q_k^2\beta_l + \frac{(q_k - p_k)}{\sqrt{2p_k q_k}}(R_{kl} - r_{kl})2p_k q_k \beta_l + \frac{2q_k}{\sqrt{2p_k q_k}}(2R_{kl}-1)p_k^2\beta_l\right]^2 = -2p_k q_k(1 - r_{kl} - R_{kl})^2\beta_l^2$$

is half the mathematical expectation of the regression coefficient that is derived from the squared difference.
